# Development of SARS-CoV-2 specific IgG and IgA antibodies in serum and milk with different SARS-COV-2 vaccines in lactating women

**DOI:** 10.1186/s13006-022-00536-y

**Published:** 2023-01-11

**Authors:** Carolina Lechosa-Muñiz, María Paz-Zulueta, Juan Irure-Ventura, Jose Manuel Mendez-Legaza, Rocío Cuesta González, Inés Gómez-Acebo, Marcos López-Hoyos, Javier Llorca, María Jesús Cabero-Pérez

**Affiliations:** 1 Departamento de Enfermería, Universidad de Cantabria, Santander, Spain; 2 Breastfeeding Coordinator, IBCLC, Hospital Universitario Marqués de Valdecilla, Cantabria, Spain; 3 IDIVAL- Grupo de Investigación en Derecho Sanitario y Bioética, GRIDES, Cantabria, Spain; 4 Department of Immunology, Hospital Universitario Marqués de Valdecilla, Santander, Spain; 5 Department of Microbiology, Hospital Universitario Marqués de Valdecilla, Cantabria, Spain; 6 Department of Pediatrics, Hospital Universitario Marqués de Valdecilla, Cantabria, Spain; 7 Departamento de Ciencias Médicas y Quirúrgicas. Universidad de Cantabria, Santander, Spain; 8 IDIVAL, Cantabria, Spain; 9 CIBER Epidemiology and Public Health (CIBERESP), Madrid, Spain; 10 Laboratory, Molecular Biology Department, University of Cantabria, Santander, Spain; 11 Red de Salud Materno Infantil y del Desarrollo – SAMID. Instituto de Salud Carlos III, Madrid, Spain

**Keywords:** Adverse effects, SARS-COV-2 vaccine, Breastfeeding, Breast milk, Maternal immunity, Neonatal immunity, Antibodies

## Abstract

**Background:**

Our main objective was to determine the evolution of IgG and IgA antibodies directed against SARS-CoV-2 protein S in the blood of lactating women and in breast milk.

**Methods:**

A cohort of 110 uninfected and vaccinated breastfeeding women was followed-up for 6 months at the Marqués de Valdecilla University Hospital, Spain, in 2020. An additional group of 23 breastfeeding mothers who had no previously documented infection and had not been vaccinated against SARS-CoV-2 were included as a control group. The antibodies in blood and breast milk and their evolution at 6 months post-vaccination were analysed.

**Results:**

One hundred ten breastfeeding mothers were included; 70 women (63.6%) were vaccinated with two doses of BNT162b2, 20 women (18.2%) received two doses of mRNA-1273, and 20 women (18.2%) received a single dose of ChAdOx1-S. No evidence of differences between concentrations of antibodies was found according to the type of vaccine, with the exception of serum IgA antibodies, which was higher in women vaccinated with mRNA-1273: mean [95%CI]: 0.05 AU/mL [0.03,0.06] with mRNA-1273, 0.02 AU/mL [0.01,0.03] with BNT162b2 and 0.01 AU/mL [0.00,0.03] with ChAdOx1-S, ANOVA *p* value = 0.03. The lack of difference between vaccines was also found when anti-S1 specific IgG in serum and breast milk were measured.

**Conclusions:**

In lactating women vaccinated against COVID-19, anti-SARS-CoV-2 antibodies can be detected in both serum and breastmilk 6 months after receiving the second dose, although their concentrations decreased when compared with concentrations reached immediately after vaccination.

**Supplementary Information:**

The online version contains supplementary material available at 10.1186/s13006-022-00536-y.

## Background

Breastfeeding is the gold standard for infant feeding. Exclusive breastfeeding is recommended for the first 6 months of life, continuing with complementary foods for at least the first year of life and beyond. These recommendations are strongly supported by multiple medical and professional childcare organizations, including the American Academy of Pediatrics (AAP) [1], the American Academy of Family Physicians (AAFP) [2], the American College of Obstetricians and Gynecologists (ACOG) [3], the World Health Organization (WHO) [4] and the Canadian Pediatric Society (CPS) [5], the American Academy of Breastfeeding (ABM) [6] and the Spanish Association of Pediatrics [7] based on short- and long-term benefits for mother and child. Suboptimal breastfeeding is associated with an increased risk of infant and child morbidity and mortality, and an increased risk of certain chronic conditions [8–12].

With the initiation of vaccination campaigns against SARS-CoV-2, many questions were raised about its compatibility with breastfeeding. Vaccination is now recommended for all postpartum individuals, including those who are breastfeeding. Mothers should not discontinue breastfeeding if they choose to be vaccinated. Although breastfeeding women were not included in the initial large vaccine trials, the available vaccines are unlikely to pose a risk to the breastfeeding infant. These vaccines do not contain infectious viruses and the minimal amount of vaccine that passes into breast milk [13, 14] and is then ingested by the infant is likely to be inactivated by the infant’s digestive system. In addition, maternal antibodies to SARS-CoV-2 induced by maternal vaccination can pass into breast milk and may offer passive protection to the infant [15–20].

Our main objective was to determine the evolution of IgG and IgA antibodies directed against SARS-CoV-2 protein S in the blood and breast milk of lactating women vaccinated with different vaccines.

### Design and setting

**Fig. 1:**
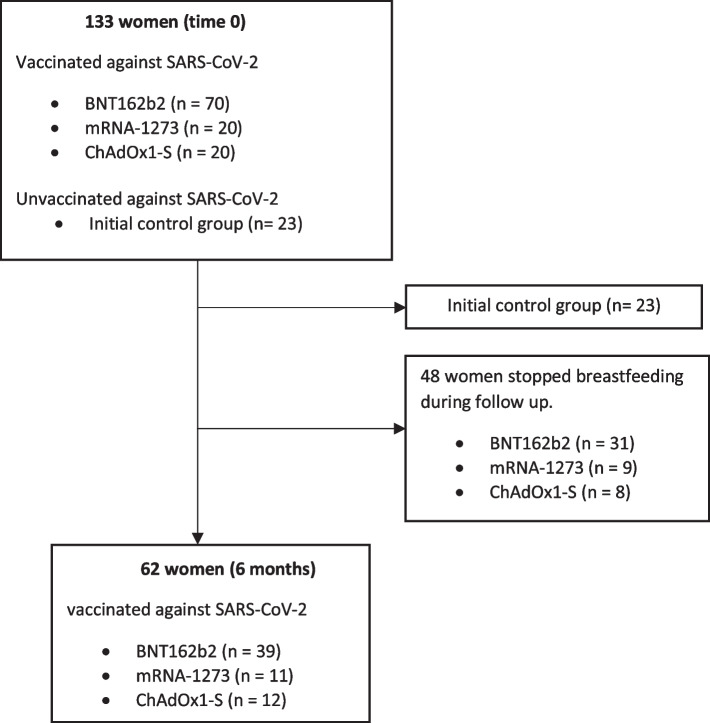
Participants & samples

A cohort study was conducted, including 110 uninfected, vaccinated breastfeeding mothers and an initial control group of 23 additional infants who had no previously documented infection and had not been vaccinated against SARS-CoV-2. Once baseline values were established in the unvaccinated uninfected unvaccinated women (where the arithmetic mean was 0.02 AU/mL [range: 0.00–0.04] in the determination of antibodies in breast milk [20]) it was not necessary to further analyze this control group and they were excluded from the 6-month follow-up. All lactating women who received both doses of the vaccine were included in the study, together with women vaccinated with ChAdOx1-S, who received a single dose. It is of note that vaccination with ChAdOx1-S was halted during the study because of the appearance of severe episodes of vaccine-induced immune thrombotic thrombocytopenia; for this reason, the second dose for breastfeeding mothers who received the first dose of ChAdOx1-S was delayed. Thus, the women recruited in our study had received two doses of either mRNA-based vaccines (BNT162b2 or mRNA-1273) or just one dose of ChAdOx1-S vaccine. Recruitment of breastfeeding mothers into the study was informed by a mass institutional mailing. All women interested in participating underwent a structured interview for data collection and collection of blood and breast milk samples after informed consent. The study was conducted at the Hospital Universitario Marqués de Valdecilla, Santander, Spain. The initial sample recruitment period was from 1 April 2021 to 30 April 2021 with a follow-up after 6 months. Our previously published study [20] refers to the initial period from April 2021 in which the safety of the vaccines and the determination of the antibody production response 1 month after vaccination are evaluated. In this new study, we report on the six-month follow-up, carried out in October 2021. During the follow-up period, 48 women stopped breastfeeding, which is why the sample at 6 months was reduced to 62 women. Blood and milk samples were taken 1 month and 6 months after the last vaccination dose. See Fig. 1 of participants & samples.

### Sources of information and data gathering

The main study variables were: mothers’ age, educational level, employment, medical history of gynecological interest, current pharmacological treatment, type of breastfeeding (exclusive or mixed with formula), infant’s age, type of vaccine received (BNT162b2, mRNA − 1273, or ChAdOx1-S) and batch, dates of vaccination, adverse effects in the mother (none, local pain, fever, malaise, lymphadenopathy, headache, nausea and others) and adverse effects in the infant after each dose and history of SARS-CoV − 2 infection.

### Collection and processing of breast milk and serum samples

The preservation and processing of the serum and breast milk samples followed the same conditions as the initial samples collected in the previous study period [20]. In the initial period, serum and breast milk samples were collected from all 110 lactating mothers in the study. Samples were collected 30 days after the second dose of vaccine (mean 30.3 days, SD 0.56), irrespective of the type of vaccine received. In the second recruitment period during the month of October 2021, serum and breast milk samples were once again collected from 62 of the 110 lactating mothers from the initial period. Following the same processing conditions, at least 5 mL of venous blood without anticoagulants and 1 mL of milk were collected. The blood sample was centrifuged at 3000 rpm (rpm) for 10 min at room temperature and the sera were divided into aliquots in cryogenic vials and stored at − 20 °C until use. Breast milk was centrifuged at 2000 rpm at 4 °C for 25 min and the supernatant was divided into aliquots in cryogenic vials and stored at − 20 °C until use. Prior to processing, breast milk samples were thawed, centrifuged at 2000 rpm for 15 min, fat was removed, and the supernatant was transferred to a new tube. Centrifugation was repeated twice to ensure removal of all cells and fat.

All serum and breast milk samples were tested in parallel on two different SARS-CoV-2 antibody test platforms, which are described in detail below.

### Detection of IgG and IgA antibodies by ELISA

The detection of IgG and IgA isotype antibodies present in serum derived from venous blood samples and breast milk samples against SARS-CoV-2 was carried out by ELISA following the protocol published by IrsiCaixa [21] and used in several publications as described below. A list of reagents and consumables and their details can be consulted in this reference [21] for its reproducibility by other interested groups.

Briefly, serum samples were pre-diluted 1:100 using phosphate buffered saline (PBS) and breast milk samples were used without any dilution.

Nunc MaxiSorp 96-well plates (Thermo Fisher Scientific, Waltham, MA, USA) were coated using optimized concentrations of the capture antibody (2 μg/mL) (MA1–21315, Thermo Fisher Scientific) diluted in PBS for 17–24 hours at 4 °C. The capture antibody used was a His Tag monoclonal antibody (HIS.H8). The washing and blocking cycle was performed with 1x PBS + 1% bovine serum albumin (BSA) for 2 h at room temperature. After a further wash cycle, S2 antigen + RBD (Sino Biological, Beijing, China) diluted in blocking buffer was added to one half of the plate and blocking buffer was added to the other half, followed by incubation for 17–24 hours at 4 °C. Specifically, the antigens used were His recombinant proteins (SARS-CoV-2 (2019-nCoV) Spike S2 ECD-His Recombinant Protein, and SARS-CoV-2 (2019-nCoV) Spike RBD-His Recombinant Protein). Serum and breast milk samples were added and incubated for 1 h at room temperature. Subsequently, incubation with peroxidase-conjugated anti-IgG and anti-IgA detection antibodies (Jackson Immunoresearch, West Grove, PA, USA) was carried out for 30 min at room temperature. Specifically, the detection antibodies used were peroxidase AffiniPure F(ab´)_2_ fragment goat anti-human IgG, Fcγ fragment specific; peroxidase AffiniPure F(ab´)_2_ fragment goat anti-human IgM, Fc5μ fragment specific; and peroxidase AffiniPure goat anti-human IgA, α chain specific. Finally, the substrate solution and the corresponding stop solution were added. The resulting absorbance was determined at 492 nm spectrophotometrically using the plate reader Infinite M200 (Tecan Magellan™). The specific signal associated with each sample was calculated by background subtraction as follows: AU specific signal = OD (+Ag) - OD (−Ag), where OD (+Ag) is the optical density (OD) obtained in the wells containing the antigens and the OD (−Ag) is the OD obtained in the control wells where no antigen was added. Arbitrary units were used because the units were defined by a measurement procedure that is not traceable to an international unit or an international certified reference material [22].

### Detection of anti-S1 IgG antibodies by chemiluminescent microparticle immunoassay (CLIA)

Serum and breast milk samples were tested in parallel with the Alinity SARS-CoV-2 IgG II Quant Assay by the Alinity i immunoassay system (Abbott, Abbott Park, IL, USA) for the determination of IgG antibodies directed against SARS-CoV-2 S1 protein (RBD). This assay is validated for application in human serum and plasma, although several studies have already applied this assay in breast milk samples and our working group has previously referenced it [20]. For the interpretation of the value as a positive result in the determination of antibodies, a value above 50 AU/mL in serum samples was considered positive, following the manufacturer’s indications. For the determination of antibodies in breast milk, the arithmetic mean of the values obtained in the milks of the control group (unvaccinated lactating mothers) was subtracted from the analytical result of each sample. This control group consisted of a total of 23 unvaccinated and uninfected lactating mothers, also referred to in our previously published study [20]. In this manner, possible analytical interferences in the determination of antibodies in breast milk are eliminated, as the sample is heterogeneous in nature.

### Statistical analysis

Comparisons of antibody concentrations between all three vaccines was performed using ANOVA. The correlation between different types of antibodies at recruitment and at the 6 months follow-up was studied with the linear correlation coefficient, stratifying for the administered vaccine. Differences in antibody concentration between time 0 and time 6 months was carried out with the paired t tests. We considered the possibility of adjusting for a number of confounders. However, age, body mass index and weight gain in pregnancy were far from associated with the type of vaccine, whereas nationality, educational level, occupational situation and method of fertilization had little or no variability; therefore, we discarded their use for adjustment. All statistical analyses were performed using the Stata 16/SE package (Stata Corp, College Station, Tx, US).

## Results

In total, 110 women were recruited; their characteristics have been described elsewhere [20]. In brief, they had been vaccinated against SARS-CoV-2 with two doses of BNT162b2 (*n* = 70), mRNA-1273 (*n* = 20) or ChAdOx1-S (*n* = 20). Most women had university studies (*n* = 103, 94%) and were health care workers (*n* = 85, 77%); 61 (56%) were primiparous, 87 (79%) had no diagnosis of any chronic disease, 94 (86%) were not receiving any medical treatment at recruitment. During the follow-up period, 48 women stopped breastfeeding, which is why the sample at 6 months was reduced to 62 women. The mean age of these women was 36.9 ranging from 26.7 to 46.5 years. Of these, 58 women (93.5%) had a university education and 49 women (79%) worked in the health sector. In relation to the vaccines, 39 women (62.9%) were vaccinated with two doses of BNT162b2, 11 women (17.7%) with two doses of miRNA-1273, and 12 women (19.4%) with one dose of ChAdOx1-S. The remaining characteristics of the sample are detailed in Supplementary Table 1.

**Fig. 2:**
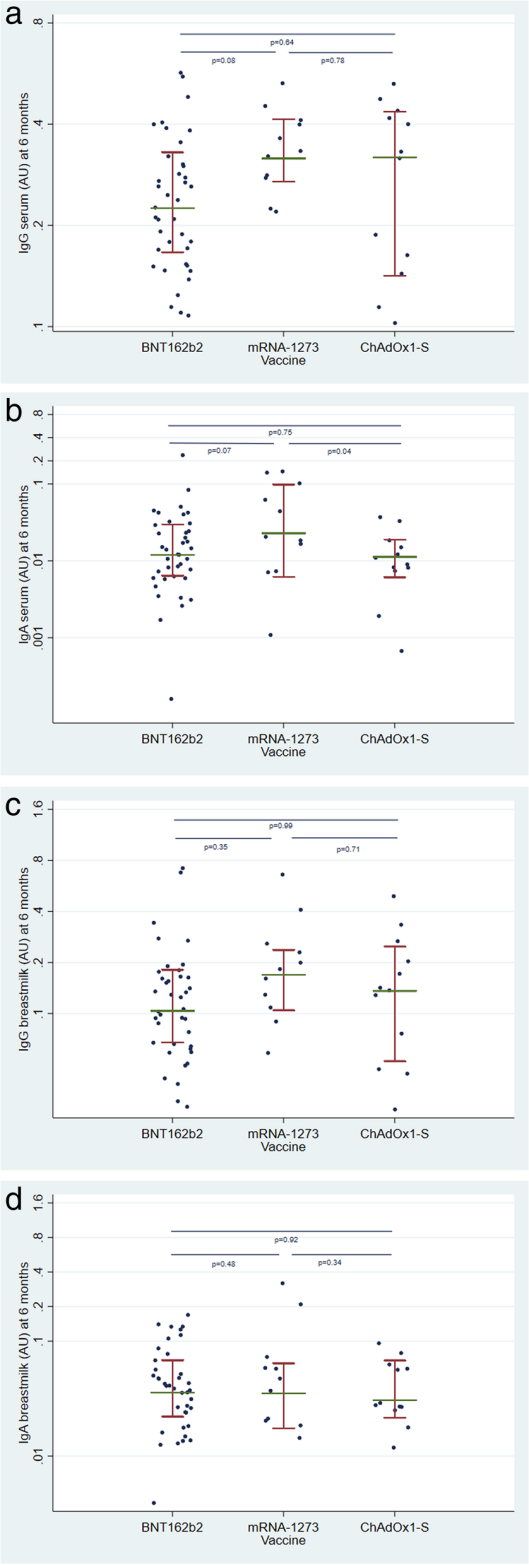
Concentration of anti-SARS-CoV-2 antibodies in breastmilk 6 months after vaccination, according to the type of vaccine. **a** IgG in serum, **b** IgA in serum, **c** IgG in breastmilk, **d** IgA in breastmilk

Concentrations of antibodies in serum and breast milk at 6 months after recruitment appear in Table 1 and Fig. 2. As described in the study design, for the determination of breast milk IgG antibodies, the arithmetic mean of the control group (unvaccinated lactating mothers) was subtracted from the analytical result of each sample. This control group consisted of a total of 23 lactating mothers, where the arithmetic mean was 0.02 AU/mL [range: 0.00–0.04]. In this way, possible analytical interferences in the determination of antibodies in breast milk were avoided.

**Table 1:** Concentrations of IgG and IgA anti-SARS-CoV-2 antibodies in serum and breast milk 6 months after recruitment, according to the type of vaccine received

Antibody	Vaccine	n	Range	AU Mean (95% CI)	F	df	Anova ***p*** value
**IgG serum (AU/mL)**	**BNT162b2**	39	0.10, 0.52	0.25 (0.22, 0.29)	2.35	2,59	0.10
	**mRNA-1273**	11	0.21, 0.53	0.34 (0.27, 0.41)			
	**ChAdOx1-S**	12	0.11, 0.48	0.29 (0.23, 0.36)			
**IgA serum (AU/mL)**	**BNT162b2**	39	0.00, 0.16	0.02 (0.01, 0.03)	3.73	2,59	0.03
	**mRNA-1273**	11	0.00, 0.14	0.05 (0.03, 0.06)			
	**ChAdOx1-S**	12	0.00, 0.03	0.01 (0.00, 0.03)			
**IgG breastmilk (AU/mL)**	**BNT162b2**	39	0.03, 0.60	0.15 (0.10, 0.19)	1.15	2,59	0.32
	**mRNA-1273**	11	0.05, 0.66	0.22 (0.14, 0.30)			
	**ChAdOx1-S**	12	0.03, 0.40	0.16 (0.08, 0.24)			
**IgA breastmilk (AU/mL)**	**BNT162b2**	39	0.00, 0.16	0.05 (0.04, 0.07)	1.28	2,59	0.29
	**mRNA-1273**	11	0.01, 0.32	0.08 (0.04, 0.11)			
	**ChAdOx1-S**	12	0.01, 0.09	0.04 (0.01, 0.07)			

*Abbreviations*: *AU* arbitrary units, *CI* confidence interval

We did not find evidence of differences between concentrations of antibodies according to the type of vaccine, except for the level of serum IgA, which was higher in women vaccinated with mRNA-1273 (mean 0.05 [95% CI: 0.03, 0.06] vs. 0.02 [0.01, 0.03] in vaccinated with BNT162b2 and 0.01 [0.00, 0.03] in vaccinated with ChAdOx1-S; ANOVA *p*-value = 0.03). The lack of difference between vaccines was also found when anti-S1 specific IgG in serum and breast milk were measured (Table 2).

**Table 2:** Concentrations of anti-S1 antibodies (IgG) in serum and breast milk according to the type of vaccine received

Antibody	Vaccine	N	Range	Mean (SD)	F	df	***p***
**Anti-S1 IgG serum (AU/mL)**	**BNT16b2**	39	71–22,988	1604.69 (4235)	0.16	2,59	0.85
	**mRNA-1273**	11	372–3093	1381.92 (757)			
	**ChAdOx1-S**	12	118–7040	2178.62 (2431)			
**Anti-S1 IgG breast milk (AU/mL)**	**BNT16b2**	39	0–149	13.62 (28)	0.13	2,59	0.88
	**mRNA-1273**	11	0–34	11.09 (10)			
	**ChAdOx1-S**	12	0–54	16.09 (17)			

*Abbreviations*: *AU* arbitrary units, *SD* standard deviation, *df* degrees of freedom

The linear correlation between concentrations of antibodies at recruitment and 6 months later is displayed in Table 3. The main patterns emerging from this correlation coefficients are (1) mild to high correlations between all antibodies at 6 months in women vaccinated with BNT162b2 or mRNA-1273- as indicated by red and medium red colors in the upper-right quadrant of the heatmap-, however, weak correlations -indicated by pale red color- were found in those vaccinated with ChAdOx1-S. (2) Weakly positive (pale red squares) or even weakly negative (pale blue squares) correlations between antibodies measured at recruitment and those measured at 6 months, are also shown irrespective of the vaccine received.

**Table 3:**
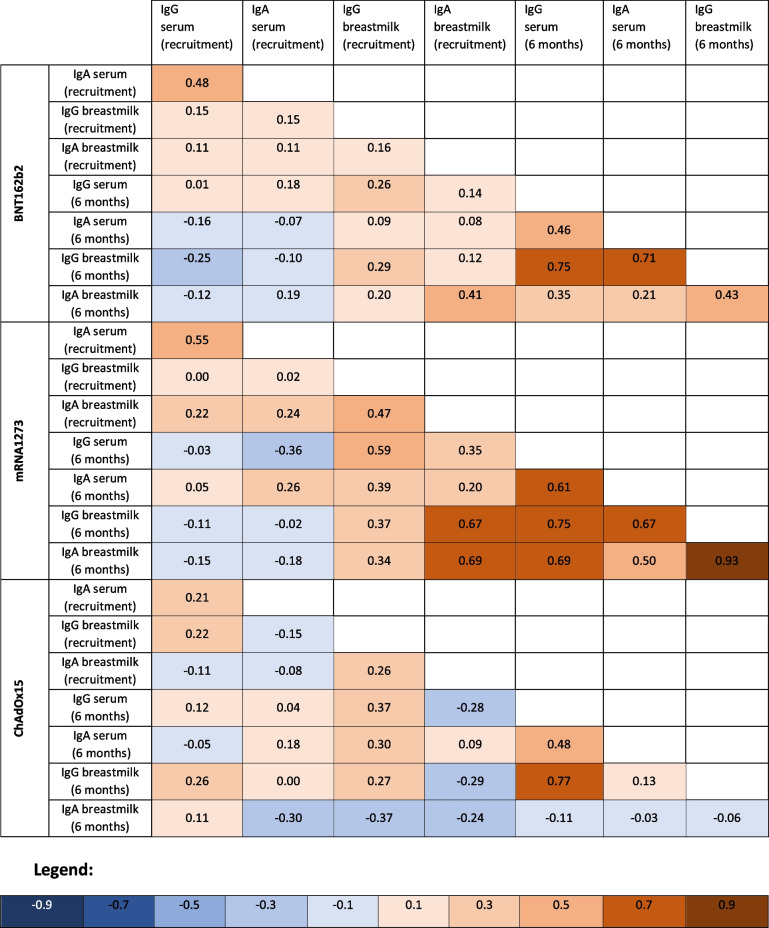
Linear correlation coefficients between concentrations of IgG and IgA anti-SARS-CoV-2 antibodies in serum and breastmilk, according to the type of vaccine received

Table 4 demonstrates the decrease in antibody concentrations from time 0 to time 6 months in women vaccinated with BNT162b2 or mRNA-1273. Altogether, the decrease seems to be lower in those receiving mRNA-1273 and even absent in serum IgG concentration (mRNA-1273).

**Table 4:** Differences between time 0 and 6 months in concentrations of IgG and IgA anti-SARS-CoV-2 antibodies in serum and breast milk according to the type of vaccine received

Antibody	Vaccine	n	Time 0 AU Mean ± SD	6 months AU Mean ± SD	Paired t test	df	p
**Serum IgA (AU/mL)**	**BNT162b2**	39	0.00–0.49	0.00–0.16			
			0.14 ± 0.08	0.02 ± 0.03	7.74	38	< 0.01
	**mRNA-1273**	11	0.004–0.42	0.00–0.15			
			0.14 ± 0.14	0.05 ± 0.05	2.22	10	0.05
**Serum IgG (AU/mL)**	**BNT162b2**	39	0.09–0.60	0.10–0.52			
			0.31 ± 0.12	0.25 ± 0.11	2.00	38	0.05
	**mRNA-1273**	11	0.15–0.51	0.21–0.53			
			0.31 ± 0.08	0.34 ± 0.10	0.67	10	0.52
**Breast milk IgA (AU/mL)**	**BNT162b2**	39	0.00–0.56	0.00–0.16			
			0.12 ± 0.13	0.05 ± 0.04	3.42	38	0.00
	**mRNA-1273**	10	0.00–0.25	0.02–0.32			
			0.12 ± 0.09	0.08 ± 0.10	1.73	10	0.11
**Breast milk IgG (AU/mL)**	**BNT162b2**	39	0.07–0.619	0.03–0.60			
			0.40 ± 0.11	0.15 ± 0.13	11.40	38	< 0.01
	**mRNA-1273**	11	0.23–0.60	0.05–0.66			
			0.46 ± 0.09	0.22 ± 0.17	4.80	10	0.00

*Abbreviations*: *AU* arbitrary units, *SD* standard deviation, *df* degrees of freedom

## Discussion

According to our results (Table 2), the presence of antibodies in both serum and breast milk continues after 6 months of vaccination against SARS-CoV-2, although concentrations have decreased. Serum and breast milk IgG and IgA at 6 months were correlated with each other in women vaccinated with messenger-RNA based vaccines, but not in those vaccinated with ChAdOx1-S. The correlation between antibody concentrations immediately after vaccination and antibody concentrations 6 months later was weak, eventually implying that the immediate response is not a good subrogate of the long-term response.

COVID-19 mRNA vaccines are immunogenic in pregnant and lactating women [17, 20], eventually leading to clinical efficacy [23]. Most studies on persistence of antibodies after vaccination have limited their follow-up to few weeks after completing the vaccination schedule. In this regard, Yeo et al. [24] found serum and breastmilk antibodies were moderately correlated 7 days after dose 2 of BNT162b2. Golan et al. [13] reported a decrease in breastmilk IgA -but not IgG- concentrations 4–10 weeks after the second dose; furthermore, the correlation between IgG and IgA antibody concentrations in breastmilk they found after the first dose disappeared after the second dose. Scrimin et al. [25] showed that antibodies in serum were lower than those in breastmilk, although their data included only 42 women and the period for obtaining biological samples extended from 20 days to 4 months after the second dose of vaccine. Charepe et al. (2021) [26] found a very week correlation (*R* = 0.12) between serum and breast milk IgA after the first dose, although this was moderate (*R* = 0.76) after the second dose. They did not perform any further follow-up.

The presence of anti-SARS-CoV-2 IgG in serum before delivery could well play a role in protecting the neonate because IgG can be transferred transplacentally. In this regard, most children born from women vaccinated during pregnancy had detectable circulating antibodies against SARS-CoV-2 [27] and had a lower risk of hospitalization during their first 6 months of life; this maternal vaccine-associated protection was found to be higher if women had been vaccinated later in pregnancy [28]. However, the relevance of anti-SARS-CoV-2 IgA in milk has not yet been established. As IgA from breastmilk cannot reach circulation in children, their main goal is to yield local barrier immunity in children [29]. Whether or not this barrier immunity in gut mucosa is effective against SARS-CoV-2 infection is still matter for research.

### Limitations

Our study has some limitations. The most important limitation is the fact that women vaccinated using ChAdOx1-S only received a single dose; thus, their antibody concentrations were not comparable with those who were fully vaccinated with BNT162b2 or mRNA-1273. These circumstances made it impossible to measure antibody concentrations in women who were fully vaccinated with ChAdOx1-S. Secondly, most women included were health care workers, who were more exposed to SARS-CoV-2 infection than women in the general population, which could limit the generalization of our results.

Secondly, we did not determine IgA data for anti-S1. IgA data for anti-S1 is not a determination routinely used in the microbiology or immunology service for the assessment of the evolution of SARS-CoV-2 specific antibodies in serum and milk with different SARS-CoV-2 vaccines in lactating women.

Thirdly, the presence of IgM antibodies was not included in the present study because this isotype is poorly represented in breastmilk samples, and also because IgM and IgG antibodies can arise nearly simultaneously [30]. An IgM antibody response has also been described during the earliest phase of memory cell production after vaccination against SARS-CoV-2, however, these IgM memory cells then declined in the order of days [31].

We did not carry out any adjustment for confounding factors. Some such as age, body mass index and gained weight in pregnancy because of their lack of association with type of vaccine; thus, they did not accomplish the so-called second criterium for confounding [32]. Others, such as nationality, educational level or occupation, had little or no variability in our sample, which avoided them to confound the comparisons [33].

Likewise, IgG anti-S1 results have been referred to arbitrary units (BAU/mL) since at the baselin of this study, there were no standardized units of measurement for any of the commercially available serological assays. In our previous published work, the same measurements were expressed and thus a comparison has been made at 6 months to see the evolution of this antibodies over time.

WHO has recently shown a current strong correlation of the units of measurement of different serological assays with the international unit BAU/mL (BAU: Binding Antibody Units). In case of SARS-CoV-2 IgG II Quant assay, serological method used in our work for the measurement of IgG anti-S1 antibodies, the mathematical relationship between AU/mL units and he international unit BAU/mL would be the following equation: BAU/mL = 0.142*AU/mL. We thus reflect this relationship in order to better harmonize the data in order to facilitate future research in this field.

Finally, the size of the population studied is a weakness of our study, so the results obtained should be confirmed in cohorts that include more participants.

## Conclusions

In lactating women vaccinated against COVID-19, anti-SARS-CoV-2 antibodies can be detected in both serum and breastmilk 6 months after receiving the second dose, although their concentrations decreased when compared with concentrations reached immediately after vaccination. Correlations between antibodies immediately after vaccination and antibodies 6 months later were weak, which makes the initial response a non-reliable subrogate of the long-term response.

## Supplementary Information

**Additional file 1: Supplementary Table 1.** Demographic data according to vaccine use.

**Additional file 2.**

## Data Availability

Data cannot be made publicly available in compliance with the protection of patient privacy. The data are available on request from the University of Cantabria Archive (http://repositorio.unican.es/) for researchers who meet the criteria for access to confidential data. Requests may be sent to the Ethics Committee (ceicc@idival.org) or Dr. María Paz-Zulueta (maria.paz@unican.es).
