# Supplementary material for: Development of SARS-CoV-2 specific IgG and IgA antibodies in serum and milk with different SARS-COV-2 vaccines in lactating women
Source: Int Breastfeed J. 2023 Jan 11;18:3. doi: 10.1186/s13006-022-00536-y (PMC9831888; doi:10.1186/s13006-022-00536-y)
Supplement: Supplementary file 1 — Additional file 1: Supplementary Table 1. Demographic data according to vaccine use. [file 13006_2022_536_MOESM1_ESM.pdf]

Supplementary Table 1. Demographic data according to vaccine use.

Variable	Category	**BNT162b2**	**mRNA-1273**	**ChAdOx1-S**	P value
Age	Continuous	37.3±3.7	36.0±4.4	36.2±4.1	0.51
BMI	Continuous	21.7±3.3	21.9±3.1	23.5±3.8	0.30
Gained weight in pregnancy	Continuous	11.1±4.6	11.4±3.6	12.4±6.5	0.76
Nationality	Spanish	39 (100%)	11 (100%)	12 (100%)	-
Educational level	Foundation degree	3 (7.7%)	1 (9.1%)	0 (0%)	0.59
	University studies *	36 (92.3)	10 (90.9)	12 (100%)	
Occupational situation	Active	39 (100%)	11 (100%)	12 (100%)	-
Occupation	Health and care	36 (92.3%)	11 (100%)	2 (16.7%)	<0.001
	Others	3 (7.7%)	0 (0.0)	10 (83.3%)	
Fertilization method	Natural	37 (94.9%)	9 (81.8%)	12 (100%)	0.18
	Assisted	2 (5.1%)	2 (18.2%)	0	

*Women had completed their university studies according to the European Higher Education Area classification.
