# Supplementary material for: Development of SARS-CoV-2 specific IgG and IgA antibodies in serum and milk with different SARS-COV-2 vaccines in lactating women
Source: Int Breastfeed J. 2023 Jan 11;18:3. doi: 10.1186/s13006-022-00536-y (PMC9831888; doi:10.1186/s13006-022-00536-y)
Supplement: Supplementary file 2 — Additional file 2. [file 13006_2022_536_MOESM2_ESM.pdf]

Detection of SARS-CoV-2 antibodies by ELISA v1
April 29th, 2020
Jorge Carrillo,
Maria Luisa Rodriguez
Carlos Ávila

IrsiCaixa
Institut de Recerca de la Sida

Detection of SARS-CoV-2 antibodies by ELISA

Protocol by IrsiCaixa

Before starting:

If you are planning to work with sample obtained from COVID19+ individuals, it is mandatory to follow all safety guidelines and protocols. Please, before starting you must inform to your lab manager, supervisor and the Risk Assessment Committee of your institution.

1) Background:

Severe Acute Respiratory Coronavirus Type 2 (SARS-CoV-2), a new coronavirus identified in China in January 2020, has been probed as the etiological agent of the Coronavirus disease 2019 (COVID-19), a new infectious disease that came up in China at the beginning of December 2019 [Wu, F. et al. Nature (2020); Na Z. et al NEJM (2020)]. COVID-19 was declared pandemic by WHO on March 11th, 2020. Currently, more than 2.8 millions of people have got infected globally and more than 193000 deaths associated with this disease have been reported (COVID-19 Situation Reports-97, 26th April 2020, WHO). Despite that our knowledge on COVID19 is increasing daily, little is still known about the behavior and role of the humoral response in SARS-CoV-2 infected individuals. The shortage of ELISA kits and reagents for measuring SARS-CoV-2 antibodies, mainly due to break of stock, has vastly contributed to hamper our capability to study the humoral response in SARS-CoV-2 infected individuals, to identify new infections and to make a serological follow up of convalescent individuals. Here, we describe a simple ELISA protocol that allows for the detection and semi-quantification of SARS-CoV-2 antibodies in plasma samples. Because ELISA is a very common laboratory technique and many researchers have access to the necessary equipment and expertise, we think that this protocol can be easily transferred and developed in any laboratory worldwide.

2) Reagents:

For the development of the protocol, we have used the reagents described below. However, if for any reason these reagents were not available, the protocol might be performed using equivalent reagents obtained from different vendors. Equivalent plasticware, secondary antibodies, substrate or antigens can be obtained for several companies with similar quality. However, because we have not performed a head-to-head comparison, any change in the protocol should be analyzed carefully.

Detection of SARS-CoV-2 antibodies by ELISA v1
April 29th, 2020
Jorge Carrillo,
Maria Luisa Rodriguez
Carlos Ávila

IrsiCaixa
Institut de Recerca de la Sida

Reagents and consumable	Name and description	Reference	Supplier
ELISA plates	Nunc-Immuno™ MicroWell™ 96 well solid plates	M9410-1CS	Sigma-Aldrich
Bovine Serum Albumin (BSA)	MACS BSA Stock Solution	130-091-376	Miltenyi Biotech
Phosphate Buffer Saline	QCA - PBS	991095	Quimica Clinica Aplicada
Tween-20	Polyoxyethylenesorbitan monolaurate	P1379-1L	Sigma-Aldrich
SULFURIC ACID	Sulfuric acid, 95-98%, a.c.s. reagent	258105-1L-PC-M	Sigma-Aldrich
Sealing Films for ELISA	Adhesive Film for Microplates	391-1250	VWR
pipette tips			
**Antigens**			
	SARS-CoV-2 (2019-nCoV) Spike S2 ECD-His Recombinant Protein	40590-V08B	Sino Biological
	SARS-CoV-2 (2019-nCoV) Spike RBD-His Recombinant Protein	40592-V08B	Sino Biological
**Antibodies**			
Capture antibody	6x-His Tag Monoclonal Antibody (HIS.H8)	MA1-21315	ThermoFischer Scientific
Detection Antibodies	Peroxidase AffiniPure F(ab')$_2$ Fragment Goat Anti-Human IgG, Fcγ fragment specific	109-036-098	Jackson Immunoresearch
	Peroxidase AffiniPure F(ab')$_2$ Fragment Goat Anti-Human IgM, Fc5μ fragment specific	109-036-129	Jackson Immunoresearch
	Peroxidase AffiniPure Goat Anti-Human Serum IgA, α chain specific	109-035-011	Jackson Immunoresearch
**Standards and controls**			
Standard	plasma sample from a convalescent COVID-19+ individual with high titer of SARS-CoV-2 IgG, IgM and or IgA antibodies		
negative control	a pool of plasma samples from COVID19 negative individuals		
positive control	plasma sample from a COVID19+ individual		
**Buffers and solutions**			
Washing buffer	PBS 1x + 0.05% tween-20		

Blocking buffer	PBS 1x + 1% BSA		
Substrate Dilution Buffer	Phosphate-citrate buffer with sodium perborate	P4922-50CAP	Sigma-Aldrich
	Dilute one capsule in 100mL of distilled water.		
Substrate	O-phenylenediamine dydrochloride (OPD)	P8787-100TAB	Sigma-Aldrich
	For two 96 well plates, dilute one table in 11 mL of Substrate Dilution Buffer 5 minutes before use.		
Stop solution:	2N H2SO4		
**Equipment**			
single channel and multichannel pipettes			
ELISA reader			
automated microplate ELISA washer			

Protocol:

1) Coating:
Dilute the capture antibody at 2ug/mL in PBS without carrier protein. Immediately coat a 96-well ELISA microplate with 50 µL/well of the diluted capture antibody. Seal the plate and incubate overnight at 4ºC, in a wet chamber.

2) Wash the plate with 300 µL of washing buffer (x3).

3) Block plates by adding 300 µL of blocking buffer/well. Incubate at room temperature for 2 hours.

4) Wash the plate with 300 µL of washing buffer/well (x5).

5) Add 50 µL/well of antigen solution* (0.9 µg/mL of S2+0.3 µg/mL of RBD in blocking buffer) to one half of the plate and 50 µL/well of blocking buffer to the other half. Seal the plate and incubate overnight at 4ºC (alternatively, the plate can be incubated for two hours at room temperature).

Example of plate distribution:

Antigens						No antigens					

* Prepare the Antigen dilution immediately before use. Do not store diluted antigens.

6) Wash the plate with 300 µL of washing buffer/well (x5).

7) Add samples, standard and controls* (50 ul/well), seal the plate and incubate 1 hour at room temperature.

* Samples (n=16/plate), standards and controls should be prepared in advance. Samples and controls should be diluted 1/100 in blocking buffer. The standard curve should be prepared including six ½ serial dilutions of a seropositive plasma sample. The starting dilution and the number of point in the standard curve need to be setup experimentally. At least a final volume of 250 µL from each sample and standard needs to be prepared, since they are incubated with and without antigens in duplicate. That is necessary to control the intrinsic background associated with each plasma sample.
Example of plate distribution:

Antigens						No antigens					
Std 1	Std 1	sanmple 1	sanmple 1	sample 9	sample 9	Std 1	Std 1	sanmple 1	sanmple 1	sample 9	sample 9
Std 2	Std 2	sanmple 2	sanmple 2	sample 10	sample 10	Std 2	Std 2	sanmple 2	sanmple 2	sample 10	sample 10
Std 3	Std 3	sanmple 3	sanmple 3	sample 11	sample 11	Std 3	Std 3	sanmple 3	sanmple 3	sample 11	sample 11
Std 4	Std 4	sanmple 4	sanmple 4	sample 12	sample 12	Std 4	Std 4	sanmple 4	sanmple 4	sample 12	sample 12
Std 5	Std 5	sanmple 5	sanmple 5	sample 13	sample 13	Std 5	Std 5	sanmple 5	sanmple 5	sample 13	sample 13
Std 6	Std 6	sanmple 6	sanmple 6	sample 14	sample 14	Std 6	Std 6	sanmple 6	sanmple 6	sample 14	sample 14
C negative	C negative	sanmple 7	sanmple 7	sample 15	sample 15	C negative	C negative	sanmple 7	sanmple 7	sample 15	sample 15
C positive	C positive	sanmple 8	sanmple 8	sample 16	sample 16	C positive	C positive	sanmple 8	sanmple 8	sample 16	sample 16

8) Wash the plate with 300 µL/well of washing buffer (x5).

9) Add 50 µL/well of the detection antibody diluted in blocking buffer* (anti-IgG 1/20000; anti-IgM 1/10000, anti-IgA 1/20000). Incubate for 30 minutes at room temperature.
*Dilution factors needed may be different according to each batch of secondary antibody. The final concentration should be determined empirically.

10) Wash the plate with 300 µL/well of washing buffer (x5).

11) Add 50 µL of substrate solution to each well. Incubate at room temperature*. Avoid exposing the plate to direct light.
*The incubation times described below are illustrative and may be changed depending on the temperature and other factors, so it should be tuned empirically. All reagents should be a room temperature.
IgG: 20 minutes
IgM: 30 minutes

IgA: 20 minutes.

12) Add 50 µL of stop solution to each well. Gently tap the plate to ensure thorough mixing.

13) Determine the optical density of each well immediately, using a microplate reader set to 492nm. If wavelength correction is available, set it to 620 nm.

Analysis of results:

The specific signal associated with each samples is calculated by background subtraction as follows:

OD specific signal= Mean OD_{+Ag}- Mean OD_{-Ag}
where OD_{+Ag} is the optical density obtained in the wells containing the antigens and the OD_{-Ag} is the OD obtained in the control wells where no Ag was added.
Once the sample signal has been corrected, then the values are plotted into the standard curve to get the results. Negative values are possible. In this case, they should be considered as zero.

Standard curve should be analyzed using 4-parameter curve fit software (i.e GraphPad Prism)

By analyzing plasma samples from more than 30 uninfected individuals we have defined a cut-off of positivity of OD=0.1. However, this value should be confirmed empirically in house before starting analyzing samples.

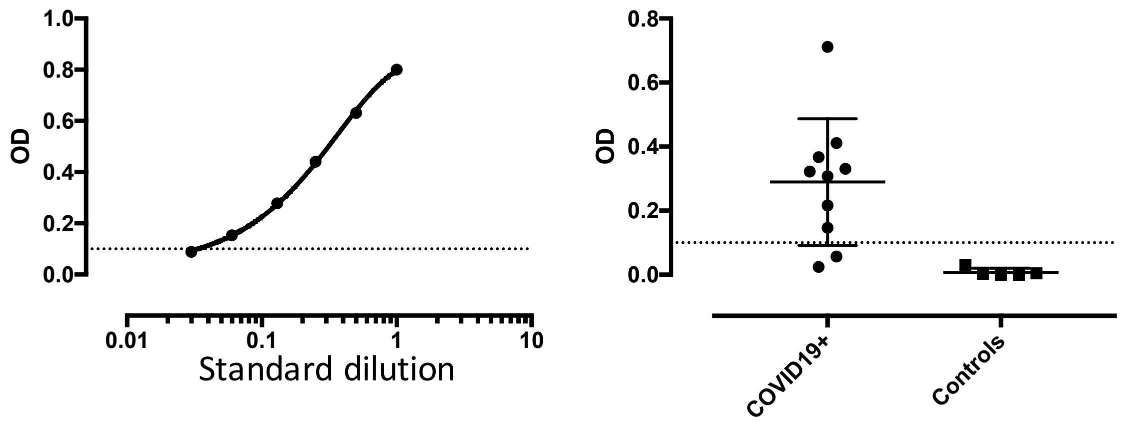

Figure 1. Representative results.
Left. A representative standard curve
Right. An example of results obtained by ELISA. Corrected OD is shown.
Dotted line indicate the positive cut-off (OD=0.1)
